# Micro-CT Imaging Reveals *Mekk3* Heterozygosity Prevents Cerebral Cavernous Malformations in *Ccm2*-Deficient Mice

**DOI:** 10.1371/journal.pone.0160833

**Published:** 2016-08-11

**Authors:** Jaesung P. Choi, Matthew Foley, Zinan Zhou, Weng-Yew Wong, Naveena Gokoolparsadh, J. Simon C. Arthur, Dean Y. Li, Xiangjian Zheng

**Affiliations:** 1 Lab of Cardiovascular Signaling, Centenary Institute, Sydney, NSW, 2050, Australia; 2 Faculty of Medicine, Sydney Medical School, University of Sydney, Sydney, NSW, 2050, Australia; 3 Australian Centre for Microscopy & Microanalysis, University of Sydney, Sydney, NSW, 2006, Australia; 4 Department of Pharmacology and Cardiovascular Institute, University of Pennsylvania, 3400 Civic Center Blvd, Philadelphia, PA, 19104; 5 Division of Cell Signaling and Immunology, University of Dundee, Dundee, DD1 5EH, United Kingdom; 6 Division of Cardiovascular Medicine and the Program in Molecular Medicine, University of Utah, Salt Lake City, UT, 84112, United States of America; BloodCenter of Wisconsin, UNITED STATES

## Abstract

Mutations in *CCM1* (aka *KRIT1*), *CCM2*, or *CCM3* (aka *PDCD10*) gene cause cerebral cavernous malformation in humans. Mouse models of CCM disease have been established by deleting *Ccm* genes in postnatal animals. These mouse models provide invaluable tools to investigate molecular mechanism and therapeutic approaches for CCM disease. However, the full value of these animal models is limited by the lack of an accurate and quantitative method to assess lesion burden and progression. In the present study we have established a refined and detailed contrast enhanced X-ray micro-CT method to measure CCM lesion burden in mouse brains. As this study utilized a voxel dimension of 9.5μm (leading to a minimum feature size of approximately 25μm), it is therefore sufficient to measure CCM lesion volume and number globally and accurately, and provide high-resolution 3-D mapping of CCM lesions in mouse brains. Using this method, we found loss of *Ccm1* or *Ccm2* in neonatal endothelium confers CCM lesions in the mouse hindbrain with similar total volume and number. This quantitative approach also demonstrated a rescue of CCM lesions with simultaneous deletion of one allele of *Mekk3*. This method would enhance the value of the established mouse models to study the molecular basis and potential therapies for CCM and other cerebrovascular diseases.

## Introduction

Cerebral cavernous malformation (CCM) is a brain vascular disease that manifests as clusters of thin dilated vessels in brain. It is common with a prevalence of 0.1–0.5% in the human population. Currently there is no drug treatment available for CCM disease and individual lesions can only be treated through surgical resection when possible [1]. Human genetic studies have identified mutations in three genes, *CCM1* (aka *Krit1*), *CCM2* and *CCM3* (aka *PDCD10*), causing CCM disease [2–6]. These genes encode non-homologous cytoplasmic proteins forming a single signaling complex. Genetic studies in the mouse and zebrafish have revealed that the CCM pathway functions in endothelial cells and is required for patterning of blood vessels and the development of embryonic heart [7–11]. In the developing heart, our recent study revealed that CCM signaling negatively controls the Mekk3 kinase cascade, *Klf2* and *Klf4* expression and *Adamts4* and *Adamts5* expression in endocardium. Deletion of *Ccm* genes in the endocardium leads to elevated Adamts activity via MEKK3-KLF signaling and causes premature degradation of cardiac matrix (jelly) and ultimately cardiac growth arrest [10]. Genetically decreasing *Mekk3* or *Klf2* gene dosage rescues the cardiac defect conferred by *Ccm* deficiency both in mice and zebrafish [10].

Various mouse models have been developed to model human CCM disease and investigate the disease-causing mechanisms downstream of CCM genes [12–15]. Among these, the most robust model is to delete conditional *Ccm* genes with Tamoxifen-inducible *Cdh5-CreERT2* or *Tie2-CreErt2* at P1 or P2 in newborn pups [13,15]. These pups display severe CCM lesion burden, from P11 onward. These mouse models have been expected to provide an invaluable foundation for pre-clinical studies to search for therapeutic agents in treating CCM diseases. However, their utility has been limited by the lack of an efficient and accurate method to quantify CCM lesion burden, a situation that has significantly restricted the application of these mouse models for pre-clinical research.

Until now, CCM lesion burden has been measured primarily using histology-based methods, an approach that is low through-put, provides an incomplete view of CCM lesions in brain, and one that is subject to investigator bias [15–17]. MRI based methods have recently been used by one research group to assess CCM lesion burden in adult mouse model [14,18]. However, a highly specialized small animal MRI instrument is required and a long scan-time (several hours to overnight) is necessary to achieve a resolution reliably identifying CCM lesions in adult mice. The ability of MRI to detect CCM lesions in neonatal mice has not been reported and resolution may limit sensitivity. Thus, a high-resolution, time and cost effective method is needed to appropriately exploit the value of mouse CCM models.

We have adopted a contrast-based micro-CT technique to image CCM lesions in the mouse model. This method provides quantitative global measurement of CCM lesion volume, accurately identifies the number and 3-D location of CCM lesions in the mouse brain, and greatly reduces the cost and time required to phenotype these animals. Micro-CT imaging of CCM lesion in mouse brain therefore provides a novel and more applicable tool to exploit CCM mouse models for mechanistic or pre-clinical studies of CCM disease. We have recently used this imaging technique to demonstrate *Mekk3*, *Klf2* or *klf4* heterozygosity prevents CCM lesion formation in *Ccm1* deficient mice [19]. In this study, we demonstrate the utility of this imaging approach by comparing the lesions in *Ccm1* and *Ccm2* deficient mice, and demonstrate that *Mekk3* heterozygosity prevents CCM lesion formation in *Ccm2* deficient mice.

## Methods

**Fig 1:**
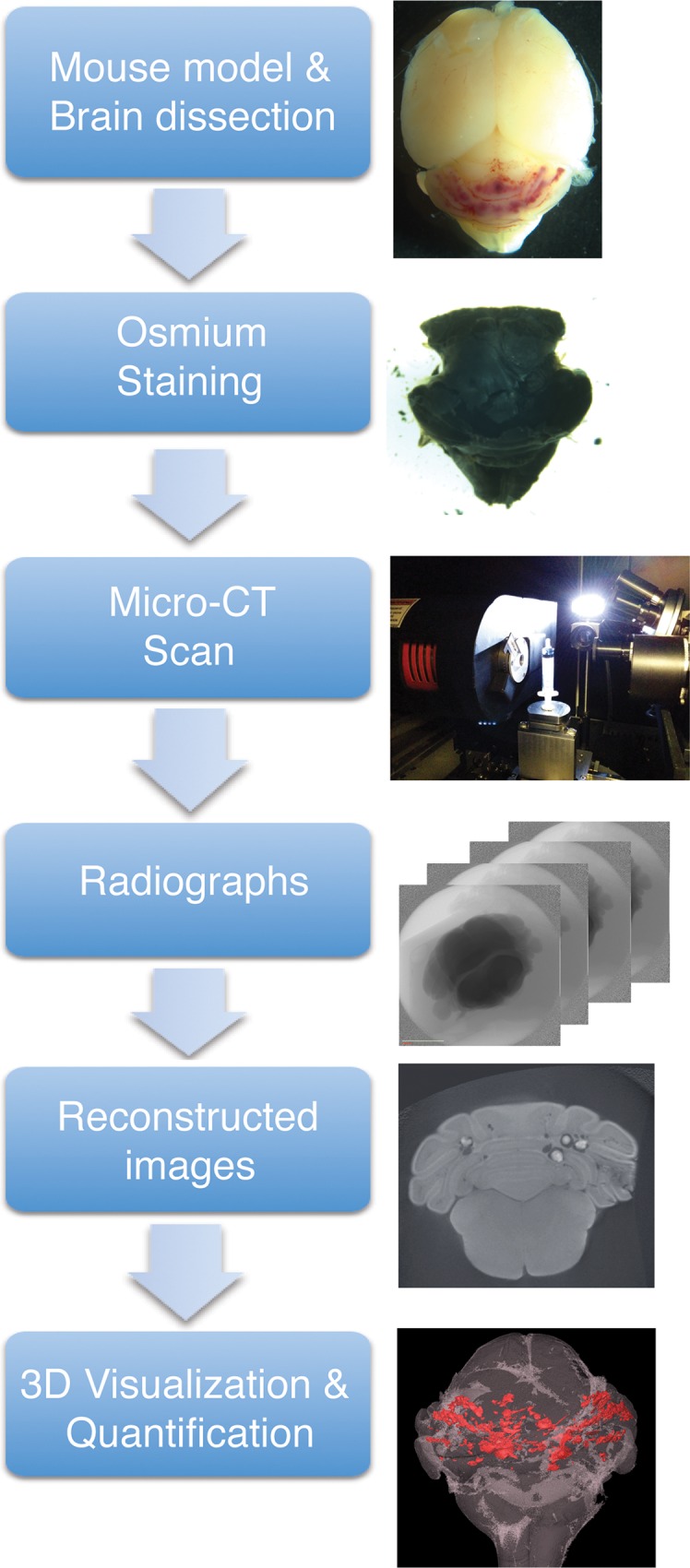
Overview of micro-CT imaging procedure. Mouse brains were dissected and fixed as described. Following the fixation, only the hindbrain was stained with OsO_4_ as a contrast staining. Osmium stained hindbrain was then scanned using micro-CT that produces series of radiographs. These radiographs were reconstructed and AVIZO software was used to produce 3D image and quantify lesions in the hindbrain.

An overview of the process is shown in Fig 1.

### Mice

All animal ethics and protocols were approved by The Sydney Local Health District Animal Welfare Committee. All experiments were conducted under the guidelines/regulations of Centenary Institute and University of Sydney.

*Cdh5-CreErt2*, *Ccm1*^*fl/fl*^, *Ccm2*^*fl/fl*^, *Mekk3*^*fl/fl*^ animals have been previously described [10,20–22]. *Cdh5-CreErt2*;*Ccm1*^*fl/+*^ mice were crossed with *Ccm1*^*fl/fl*^ to generate *Cdh5-CreErt2*;*Ccm1*^*fl/fl*^ (*Ccm1*^*iECKO*^), *Cdh5-CreErt2*;*Ccm2*^*fl/+*^ mice were crossed with *Ccm2*^*fl/fl*^ to generate *Cdh5-CreErt2*;*Ccm2*^*fl/fl*^ (*Ccm2*^*iECKO*^), and *Cdh5-CreErt2*;*Ccm2*^*fl/fl*^ mice were crossed with *Ccm2*^*fl/fl*^*;Mekk3*^*fl/+*^ mice to generate *Cdh5-CreErt2*;*Ccm2*^*fl/fl*^;*Mekk3*^fl*/+*^ (*Ccm2*^*iECKO*^*;Mekk3*^*het*^). Number of mice used in each experimental groups are outlined in each results section and figure legends.

### CCM lesion induction & Sample preparation

#### CCM lesion induction

Tamoxifen (TMX) (T5648-1G, Sigma-Aldrich) was dissolved in 100% ethanol and stored at -80°C in aliquots (TMX concentration: 50 mg/ml). On the day of use, aliquoted TMX was diluted in corn oil (C8267-500ML, Sigma-Aldrich) (4 mg/ml). Neonatal pups at postnatal day 1 (P1) were injected intragastrically with 50μl of TMX (4 mg/ml) to induce experimental CCM lesions using a 30-gauge needle. A single injection of TMX at P1 was sufficient to induce CCM lesions.

#### Sample collection

Neonatal pups at P13 were euthanized via carbon dioxide asphyxiation and intra-cardiac perfused with 3 ml of 2% paraformaldehyde in PBS solution. Whole brains were dissected and fixed 2.5% glutaraldehyde, 4% formaldehyde 0.1 M sodium phosphate buffer overnight. On the following day, hindbrains were detached and washed with phosphate buffer before contrast staining. Upon collection, a morphological image of each brain was taken as a record.

#### Contrast stains

Dissected hindbrains were incubated in 2% Osmium tetroxide (OsO_4_) (C010, Proscitech) for approximately 24 hours under gentle agitation to promote sufficient infusion of OsO_4_ into the hindbrain. Following the incubation, hindbrains were washed with distilled water three times, ten minutes each. OsO_4_ is highly toxic; hence, the safety data sheet was consulted and its handling procedures were strictly followed.

#### Sample packaging for micro-CT

OsO_4_ stained hindbrains were packaged in a 3 ml syringe (DVR-3418, Terumo) with distilled water and a small sponge on each side to secure the hindbrain and prevent it from moving during scan, and avoid direct contact with the hard syringe wall.

### Micro-CT scans

In the study, micro-CT scans were performed using an Xradia MicroXCT-400 system (Carl Zeiss XRM, USA). The hindbrain packaged in a 3 ml syringe is mounted vertically on an aluminum holder in the MicroXCT. To acquire the tomographic data sets and to optimize micro-CT scan for CCM lesions in mouse, different scanning parameters (exposure time, number of projections and beam conditions) were evaluated. The optimized scans were performed with constant source conditions of 50kV and 10W.

To assess the post-scanned specimen, the radiographic datasets were imported into hardware-based back projection reconstruction software supplied by Xradia, producing an image series of 16-bit axial slices with slice-spacing equivalent to the pixel size for each sample (isotropic voxels).

### Micro-CT scans analysis

Reconstruction of the projections resulted in an axial stack saved as 16-bit grey-scale TIFF images for further processing. Reconstruction parameters were kept constant for each scan to provide uniform grey scale factors during analysis.

The reconstructed axial slice 3D datasets were visualized using commercially available 3D rendering software, Avizo 3D image processing software (FEI Visualization Sciences Group). The 3D axial datasets were imported into Avizo 3D, and image sequences were presented in the axial (XY), frontal (YZ) and sagittal (XZ) planes. To obtain an approximately similar anatomical plane of view, digital rotation of the reconstructed image was performed on some specimens as required. The 3D reconstructions were ‘virtually sectioned’ into desired planes and 2D images from ortho-slices were captured showing the lesion distribution and brain structures.

Raw image stacks from each scanned hindbrain were analyzed for lesion identification. Lesions were identified and labeled using a greyscale intensity threshold and the shape of the feature. A region-growing segmentation algorithm included with the software (Magic Wand) was used to label individual lesions across multiple cross-sections.

Post-lesion labeling, the hindbrain image stack was isosurface (volume) rendered and overlaid with the labeled lesions in Avizo. Finally, the 3D rendered and segmented hindbrain model was cropped and rotated within the program to attain the desired spatially significant view, thus highlighting the spatial relationship of the lesions and hindbrain. It is important to point out that these changes were made post-lesion labeling, and lesion detection or visual presentation was not affected.

### Statistical analysis

All the statistical analysis in the study was done using t-tests in Graphpad Prism statistical software. Values are presented as mean±SE. Statistical significance was considered when p-value ≤0.05.

## Results

### Establishment of a micro-CT protocol to detect and analyze CCM lesions in neonatal mice

Contrast-enhancing, whole mount staining methods has been used for micro-CT imaging of soft tissues and mouse embryos [23,24], and OsO_4_ fixatives are widely used in Electron Microscopy to enhance contrast on ultrathin sections. Osmium preferentially binds to unsaturated lipid [25,26]. The brain is an organ containing a high content of unsaturated lipids. We therefore reasoned that an osmium-based contrast enhancing method would be suitable for micro-CT imaging of CCM lesions in brain.

To test whether osmium staining can be used to enhance contrast for micro-CT imaging of neonatal brain, we stained neonatal mouse brains (P13) with OsO_4_ and subjected them to high-resolution micro-CT imaging. Micro-CT is a highly technical and precise method which allows optimization of a number of settings to produce optimal images to visualize target sample. Projection count and exposure time are critical settings, as variations in either are directly proportional to the quality of the resulting image, however, they are also directly proportional to the total scan time, an important variable for procedure cost and throughput. Therefore, we have tested a number of different settings to determine the most optimal setting to scan mouse hindbrains and achieve high-resolution images in the most efficient way.

**Fig 2:**
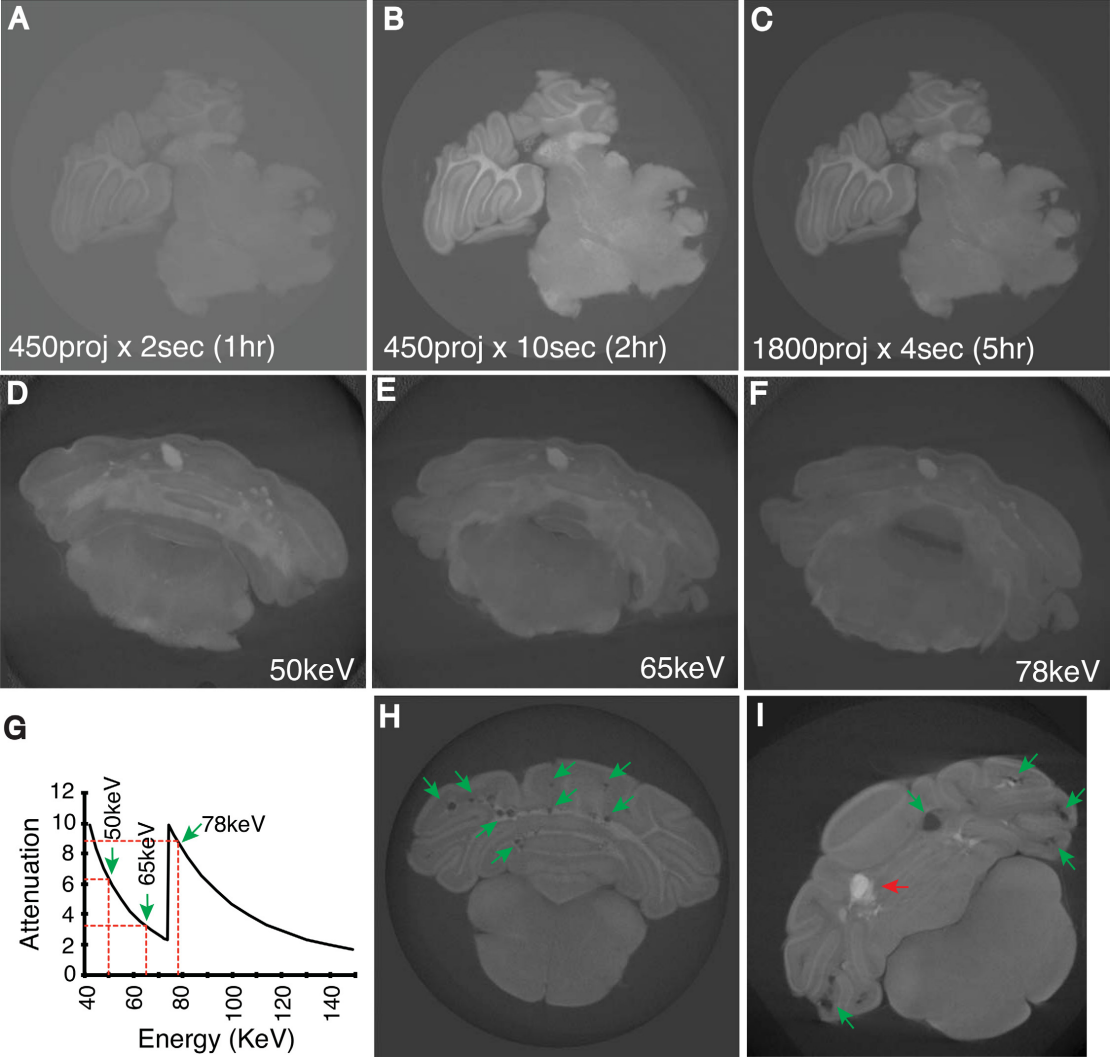
Optimization of micro-CT imaging of CCM lesions in the neonatal mouse hindbrain. Different micro-CT settings were tested to identify optimal images by which to detect CCM lesions. Initially, three different settings were tested: **A)** 450 projections x 2 seconds (1 hour scanning time), **B)** 450 projections x 10 seconds (2 hours) and **C)** 1800 projections x 4 seconds (5 hours). Furthermore, to determine the optimal beam voltage for highest attenuation of osmium, three different energy levels were tested: **D)** 50keV, **E)** 65keV and **F)** 78keV. **G)** X-ray form factor graph of osmium and the distribution of chosen energy level in the spectrum. **H-I)** Reconstructed images which detected CCM lesions (green arrows) (**H** and **I**) and distinguished blood-filled lesions (red arrows) (**I**).

We varied the number of projections and exposure times and compared image quality with the duration of the scan. The image resolution (9.5μm/pixel) was kept constant throughout the testing process. Initially, three settings were compared: 450 projection x 2second exposure time (450proj x 2sec) (Fig 2A), 450proj x 10 sec (Fig 2B) and 1800proj x 4sec (Fig 2C). The durations of the scans were approximately 1, 2 and 5 hours respectively. As expected, 1800proj x 4sec setting produced the image with best clarity followed by 450proj x 10sec. However, 1800proj x 4sec setting required scanning time of five hours. Meanwhile 450proj x 10sec setting produced sufficient image quality to determine CCM lesions in the brain. 450proj x 2sec setting resulted in unsatisfactory image quality, which was not suitable for determining CCM lesions.

Furthermore, three x-ray beam voltages were considered, taking into account osmium attenuation (Fig 2D–2F). These beam voltages were chosen based on the x-ray form factor of osmium (Fig 2G) obtained from National Institute of Standards and Technology (NIST) and equipment limitations. It was determined that using 50 keV produced the most consistent result in terms of penetration of the sample by the x-ray beam, minimisation of reconstruction artifacts, and the avoidance of any false-positive identification of lesions as a result of soft-tissue failing to adequately attenuate higher beam energies.

The number of projections and x-ray exposure time were further optimized with the goal of a total scanning duration of approximately two hours. The 720proj x 3sec setting at 50keV produced the most satisfactory image (Fig 2D). From the scan, CCM lesions were easily distinguished (Fig 2H and 2I). Importantly the scan was able to easily distinguish blood filled versus empty CCM lesions (Fig 2I). To further confirm data from 720proj x 3sec setting is not compromised by false negative detection, we compared CCM lesions (S1 Fig) identified from a single brain sample from image datasets generated at 450proj x 3sec (~1.2hr), 720proj x 3sec (~2hrs) and 1800proj x 3sec (~5hrs) setting. We found 92, 149 and 154 lesions from datasets of 450proj x 3sec, 720proj x 3sec and 1800proj x 3sec setting respectively (S1 Fig). Thus, at 720proj x 3sec setting, we were able to identify 96.8% of maximum lesion counts.

We conclude that the optimal study incorporates projection images that are integrated for 3 seconds every 0.25° over a full 180° rotation (720proj x 3sec) at 50keV for an efficient and satisfactory condition to produce micro-CT images for CCM lesion assessment in mouse, with a 9.5μm pixel resolution, and a minimum feature size of approximately 25μm [27]. All the micro-CT scans described further below were obtained using these conditions.

### 3-D Reconstruction of CCM lesions in mouse model

**Fig 3:**
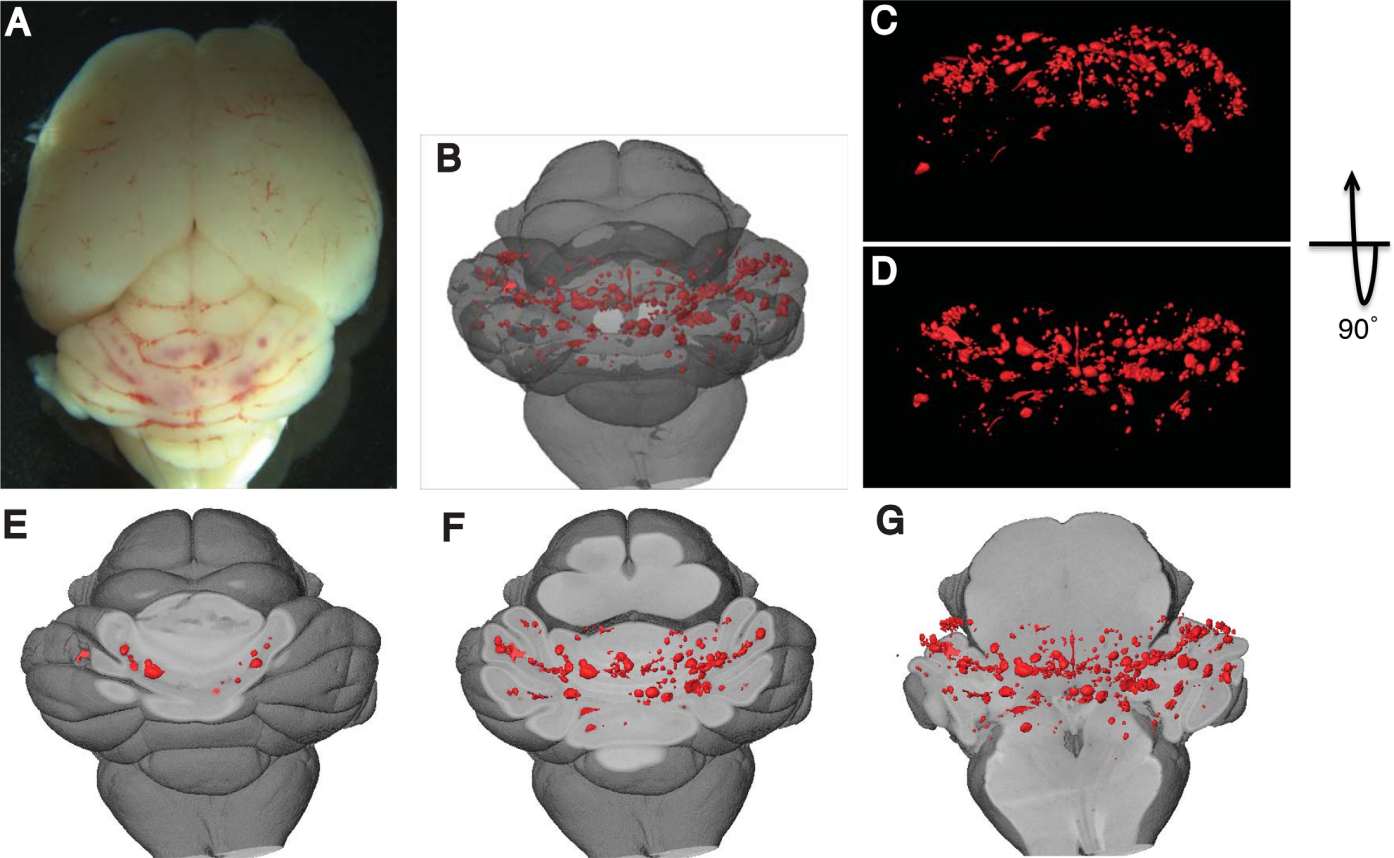
Three-dimensional images of mouse brain with CCM lesions. **A)** Macroscopic images of CCM lesions in the hindbrains of *Ccm1*^*iECKO*^ mice. **B-G)** 3-D visualization of CCM lesions at various levels and orientations in the hindbrain. CCM lesions were visualized with the reference to overall hindbrain anatomy (**B**), from the back (**C**) and above (**D**) of hindbrain, and at different depth (**E-G**) inside hindbrain (red refers to CCM lesions).

Utilizing the optimized micro-CT, we imaged CCM lesions in the hindbrains of *Ccm1*^*iECKO*^ mice (Fig 3A). Scanned x-ray images were reconstructed to produce 3D images of the mouse brain, which allowed us to visualize entire lesions in the brain parenchyma (Fig 3B–3G and S1 Video) at different depths and orientations, and to assess structure and the 3-D location of lesions in brain. Importantly, this image dataset can also be used for efficient and accurate quantification of lesion sizes and lesion numbers.

### Comparison of CCM lesions in *Ccm1*^*iECKO*^ and *Ccm2*^*iECKO*^ mice

**Fig 4:**
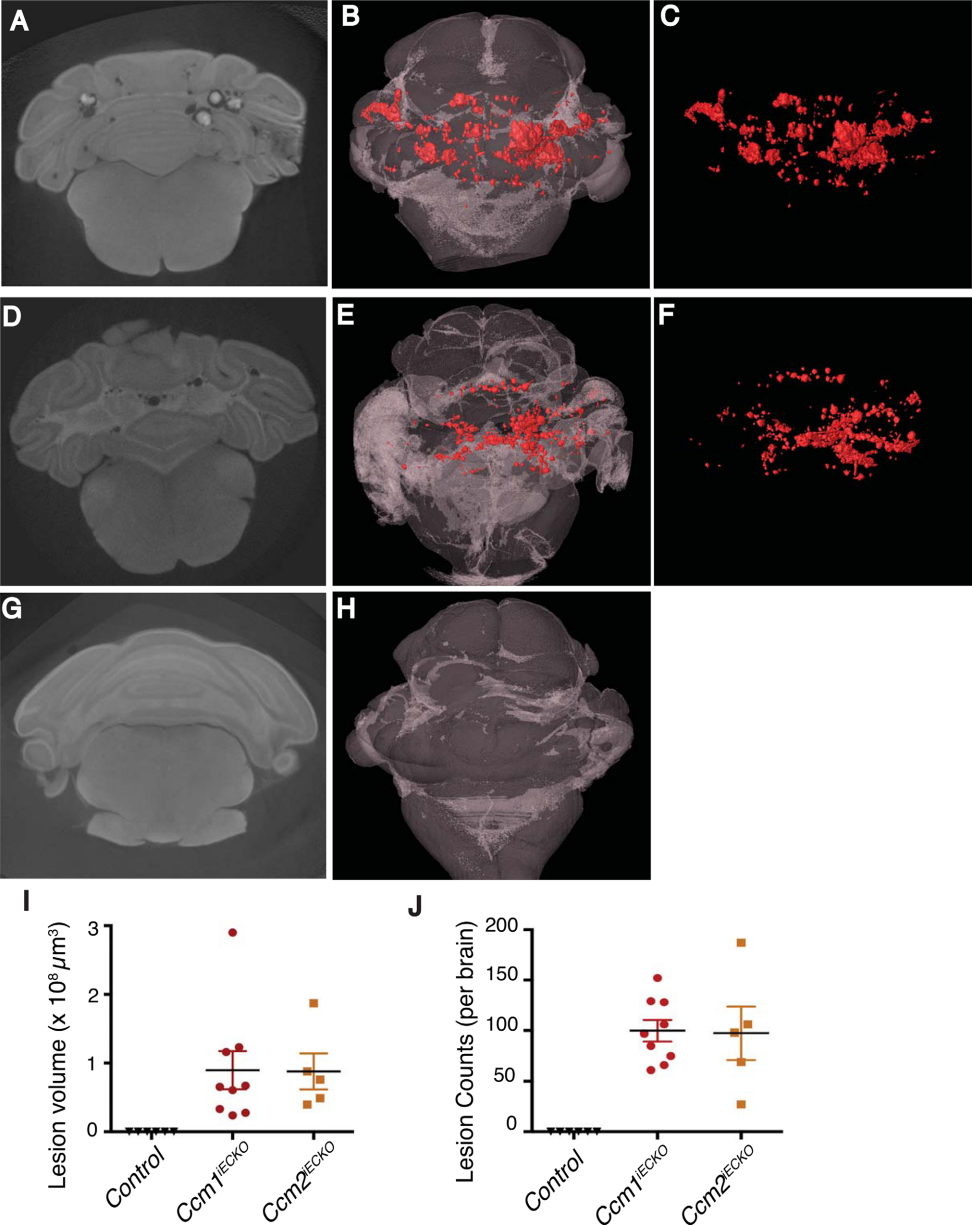
Qualitative and quantitative comparison of CCM lesions in *Ccm1i*^*ECKO*^ and *Ccm2i*^*ECKO*^ mouse models using micro-CT. **A-H)** 2-D graphs and rendered 3-D images of CCM lesions in *Ccm1i*^*ECKO*^ (**A-C**), *Ccm2i*^*ECKO*^ (**D-F**) and littermate control (*Ccm1*^*fl/fl*^ and *Ccm2*^*fl/fl*^) (**G-H**) mice (red refers to CCM lesions). **I-J**) Quantitative comparison of CCM lesions volume (**I**) and number per brain (**J**) in *Ccm1i*^*ECKO*^ (n = 9 from 4 litters) and *Ccm2i*^*ECKO*^ mice (n = 5 from 4 litters).

**Fig 5:**
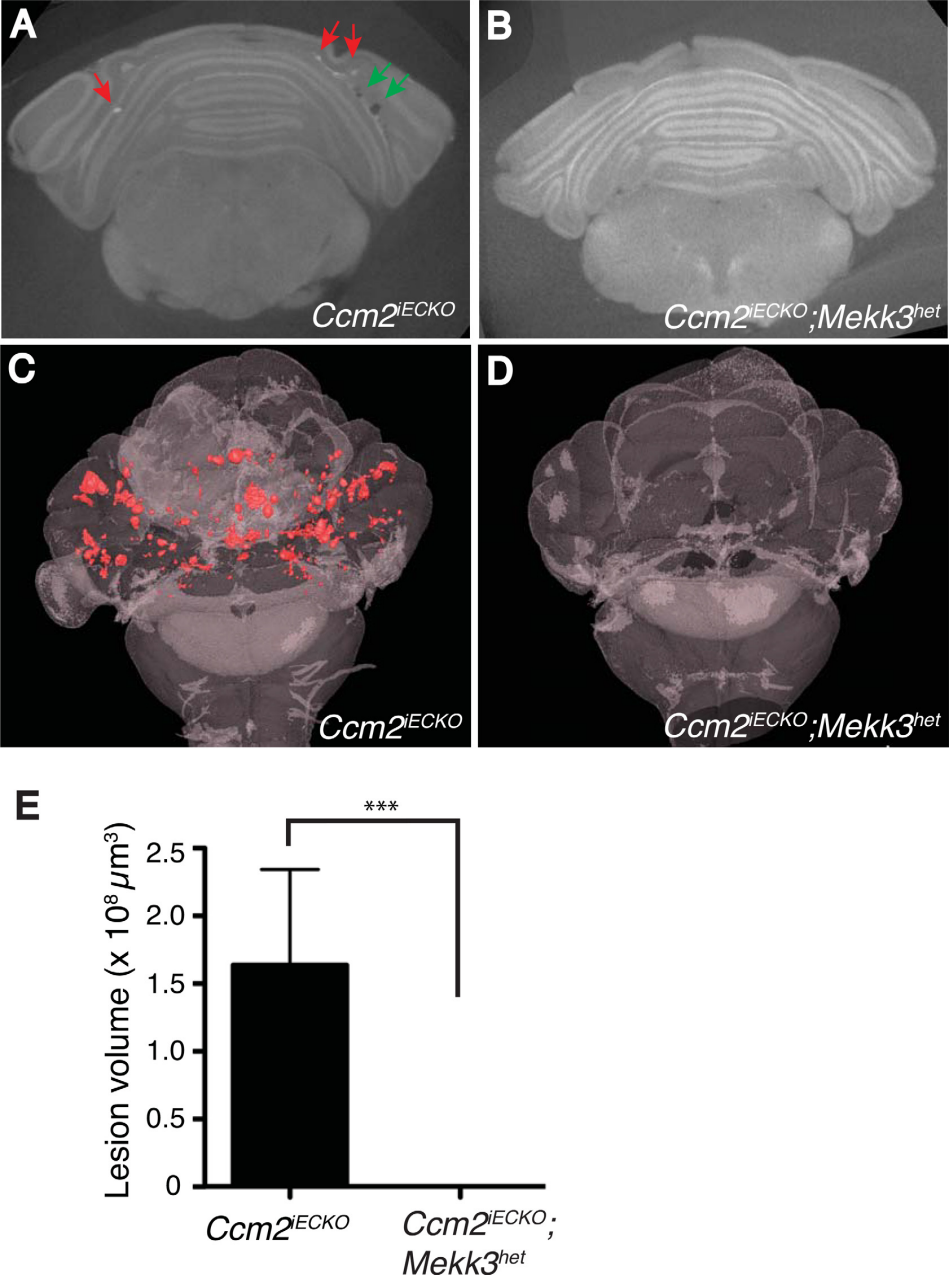
*Mekk3* heterozygous deletion rescues CCM lesions in *Ccm2i*^*ECKO*^ mice. CCM lesions were qualitatively analyzed by 3-D visualization. Representative Micro-CT scans (**A**) and 3-D projections (**C**) of *Ccm2i*^*ECKO*^ mouse and rescued mice with *Mekk3* heterozygous deletion (*Ccm2*^*iECKO*^*; Mekk3*^*het*^) (**B** and **D**). Red refers to CCM lesions. **E**) Quantitative comparison of CCM lesions volume in *Ccm2i*^*ECKO*^ (n = 5 from 4 litters) and *Ccm2i*^*ECKO*^; *Mekk3het* (n = 8 from 4 litters) mouse.

Deleting *Ccm1*, *Ccm2* or *Ccm3* gene in neonatal mice confers CCM lesions in mouse brain [13,15]. However, precise and reliable comparison of lesions (size and number) in mouse lacking different *Ccm* genes have not been reported due to the lack of an efficient technique to evaluate and quantitatively compare global CCM lesion burden in brain. Hence, we have compared lesions in 9 *Ccm1*^*iECKO*^ mice from 4 different litters (Fig 4A–4C) and 5 *Ccm2*^*iECKO*^ mice from 4 different litters (Fig 4D–4F) mice. The location and structure of CCM lesions in both models were highly similar (Fig 4B, 4C, 4E and 4F). Furthermore, to determine whether the scan detects false positives (normal blood vessels), brains of littermate control mice (3 *Ccm1*^*fl/fl*^ and 3 *Ccm2*^*fl/fl*^) treated with TMX were scanned (**G-H**). As a result, the scan failed to detect any features that appear to be lesions or normal blood vessels suggesting that the scan is optimal for detecting CCM lesions only. Lesion volume and lesion number between *Ccm1*^*iECKO*^ and *Ccm2*^*iECKO*^ mice were also similar without statistical significance (Fig 4I and 4J). These results suggest that both the *Ccm1*^*iECKO*^ and *Ccm2*^*iECKO*^ mouse models produce similar morphological CCM lesions and lesion burden.

### *Mekk3* heterozygosity rescues CCM lesion conferred by induced loss of *Ccm2* gene in endothelial cells

MEKK3 directly interacts with CCM2 to transduce CCM signaling [10,28]. Recently, we have shown that MEKK3 haploinsufficiency rescues CCM lesions in *Ccm1*^*iECKO*^ mice [19]. However, the rescue of CCM lesions in *Ccm2*^*iECKO*^ mice by decreased *Mekk3* gene dosage has not been carefully assessed. We therefore investigated the role of *Mekk3* gene dosage in CCM lesion formation in *Ccm2*^*iECKO*^ mice. As previously shown[13,15], deletion of *Ccm2* gene caused CCM lesion formation in mice (5 mice from 4 litters) (Fig 5A and 5C). Consistent with the protective role of *Mekk3* deletion in lesion formation in *Ccm1*^*iECKO*^ mice[19], we found that loss of one allele of *Mekk3* gene was sufficient to prevent lesion formation in *Ccm2*^*iECKO*^ mice (8 mice from 4 litters) (Fig 5B, 5D and 5E). In summary, our studies demonstrate that elevated MEKK3 activity is causal for CCM lesion formation in the mouse models, and suggest that partial inhibition of MEKK3 expression and/or activity is a promising therapeutic strategy to prevent and/or treat CCM disease.

## Discussion

Cerebral Cavernous Malformation (CCM) is also known as cavernoma or cavernous angioma and is a common vascular malformation that affects 0.1–0.5% of individuals [1]. CCMs can occur in sporadic or familial form. Patient prognosis is often unclear and lesions can rupture unexpectedly to cause brain hemorrhage and neurological deficits. While surgical procedures can be successful in some cases, there are currently no drugs or targeted therapeutics to prevent or halt the progression of CCM disease.

Human genetic studies have demonstrated that mutations in any of three genes (CCM1, CCM2 and CCM3) may cause CCM disease [2–6]. In the past decade, extensive work has been done in cultured cells and model organisms to elucidate the role of CCM genes is regulating endothelial cell function and cardiovascular development [7–11,29–32]. Animal models that accurately reproduce human CCM conditions have been generated in past few years, with the neonatal CCM gene deletion model providing the highest efficiency in forming lesions [12–15]. These models are potentially invaluable tools to investigate the pathogenesis of CCM disease. However, their potential has been limited by the low throughput and non-quantitative methods available to assess CCM lesion formation.

Currently, studies of CCM lesion burden rely primarily on histology-based methods. Partial sectioning of lesion-bearing brain tissue provides a general impression of CCM burden, but is not able to provide the accurate quantitation needed to compare the impact of genetic and pharmacologic studies. While a complete serial section, histological staining and reconstruction is possible, it is highly time consuming and low yield. An MRI based method has also been used to assess CCM lesions in adult mouse brains, but a long scan time (usually overnight for each sample) is required to achieve a resolution threshold necessary to identify lesions. We have tested MRI-based methods in neonatal mice, but could only detect the largest CCM lesions and were unable to visualize lesions that were easily detectable using histologic methods (not shown).

Micro-CT has the potential to image vascular lesions in the mouse brain. Microfil perfusion based micro-CT method has been used to image cerebral vascular network in adult mice. Due to the complexity of cerebral vascular system, perfusion is highly variable and dependent upon the anatomy of the vessels and skill level of the operators. Vessels in certain areas in brain are not consistently perfused, and special surgical procedures are needed to achieve consistent perfusion [33,34]. The brain vasculature in the neonatal CCM mouse model, and the sluggish or even absent circulation in CCM lesions are important technical limitations of this method.

A second potential method to adopt micro-CT for CCM lesion imaging is contrast enhancement with whole mount staining. After comparing various reagents used in micro-CT imaging and electron microscopy, we chose OsO_4_, because of its preference in binding lipid [25] and produces the most universal staining among various tissues according to previously published data of mouse embryos [23].

In our recently established micro-CT method, the sample preparation is very straightforward and does not require highly specialized training or practice. Our method can provide consistent high-resolution projections with a scan time of less than two hours. The coupling of datasets from X-ray CT scanner and the 3-D Analysis software is a powerful tool to generate a 3-D global view of CCM lesions in brain. The morphology and distribution of lesion relevant to brain anatomy can be easily observed and the total or individual lesion volume and lesion number can be retrieved readily. Comparing with histology-based 3-D reconstruction, this method is highly time and cost-effective. 3-D reconstruction from the image dataset of intact whole-mount brain tissue produces more accurate 3-D representation.

We tested this method by comparing CCM lesions formed in *Ccm1*^*iECKO*^ and *Ccm2*^*iECKO*^ animals. We found no significant difference in CCM lesion distribution, total volume and lesion number. This is consistent with molecular studies suggesting CCM1 and CCM2 function upstream in a same pathway, and human studies revealing a similar disease penetrance and history for patients with mutations in these two genes[7,9,13,35].

We have recently reported that Mekk3 is recruited to and suppressed by the CCM complex[10]. Loss of *Ccm* genes leads to increased activation of Mekk3 and its downstream transcriptional targets. Decreased *Mekk3* gene dosage prevented CCM lesion formation caused by *Ccm1* deletion[19]. In this study, we extend this observation and report that deletion of one allele of *Mekk3* in the endothelium is also sufficient to prevent CCM lesion formation caused by *Ccm2* deficiency. This further confirms *Mekk3* as a common target effector of the CCM pathway. Precise control of its activity is essential to maintain vessel integrity and prevent vessel malformation. Our studies provide additional evidence that inhibiting MEKK3 activation may be a promising therapeutic strategy for CCM disease.

Our current method is only optimized for imaging fixed mouse brain samples *ex vivo*. However, our method also has the capacity to be used for other studies involving different organs, and may serve as an exciting and novel tool for vascular malformation research in general. For pre-clinical studies of CCM and other vascular diseases, it would be advantageous to monitor lesion progression in live animals over a specified time-span. Therefore, the development of a more advanced method to image CCM lesions in live mice would further propel research efforts aimed at the development of new CCM therapeutics.

## Supporting Information

S1 Fig Verification of the optimized setting of micro-CT imaging of CCM lesions in the neonatal mouse hindbrain. To verify the micro-CT setting of 720 projections x 3 seconds is sufficient to accurately detect CCM lesions without false negatives; three different settings were tested using a single sample: **A-C)** 2-D graph generated from scan settings at 450 projections x 3 seconds (**A**), 720 projections x 3 seconds (**B**) an 1800 projections x 3 seconds (**C**). Each scan dataset was rendered and 3-D images were produced (**D-F**) and lesion numbers were calculated (**G**). As a result, 720 projections x 3 seconds and 1800 projections x 3 seconds scan detected similar lesion number suggesting that 720 projections x 3 seconds scan is sufficient and with minimum negatives. However, 450 projections x 3 second scan failed to detect all the lesions.
(DOCX)

S1 Video 3-D reconstruction of mouse brain with CCM lesions. (MOV)
