# Supplementary material for: Micro-CT Imaging Reveals Mekk3 Heterozygosity Prevents Cerebral Cavernous Malformations in Ccm2-Deficient Mice
Source: PLoS One. 2016 Aug 11;11(8):e0160833. doi: 10.1371/journal.pone.0160833 (PMC4981389; doi:10.1371/journal.pone.0160833)
Supplement: S1 Fig — To verify the micro-CT setting of 720 projections x 3 seconds is sufficient to accurately detect CCM lesions without false negatives; three different settings were tested using a single sample: A-C) 2-D graph generated from scan settings at 450 projections x 3 seconds (A), 720 projections x 3 seconds (B) an 1800 projections x 3 seconds (C). Each scan dataset was rendered and 3-D images were produced (D-F) and lesion numbers were calculated (G). As a result, 720 projections x 3 seconds and 1800 projections x 3 seconds scan detected similar lesion number suggesting that 720 projections x 3 seconds scan is sufficient and with minimum negatives. However, 450 projections x 3 second scan failed to detect all the lesions. (DOCX) [file pone.0160833.s001.docx]

**Supplemental Figure 1. Verification of the optimized setting of micro-CT imaging of CCM lesions in the neonatal mouse hindbrain.** To verify the micro-CT setting of 720 projections x 3 seconds is sufficient to accurately detect CCM lesions without false negatives; three different settings were tested using a single sample: **A-C)** 2-D graph generated from scan settings at 450 projections x 3 seconds (**A**), 720 projections x 3 seconds (**B**) an 1800 projections x 3 seconds (**C**). Each scan dataset was rendered and 3-D images were produced (**D-F**) and lesion numbers were calculated (**G**). As a result, 720 projections x 3 seconds and 1800 projections x 3 seconds scan detected similar lesion number suggesting that 720 projections x 3 seconds scan is sufficient and with minimum negatives. However, 450 projections x 3 second scan failed to detect all the lesions.
